# Effect of Chestnut (*Castanea Mollissima* Blume) Bur Polyphenol Extract on *Shigella dysenteriae*: Antibacterial Activity and the Mechanism

**DOI:** 10.3390/molecules28196990

**Published:** 2023-10-09

**Authors:** Fei Peng, Linan Chen, Xiuping Wang, Zuoqing Yu, Caihong Cheng, Yuedong Yang

**Affiliations:** 1 Hebei Key Laboratory of Active Components and Functions in Natural Products, Hebei Normal University of Science and Technology, Qinhuangdao 066000, China; feipeng1986@hotmail.com (F.P.); linanchen@163.com (L.C.); jesus_yu@163.com (Z.Y.); cch20059@126.com (C.C.); 2 Engineering Research Center of Chestnut Industry Technology, Ministry of Education, Qinhuangdao 066000, China

**Keywords:** *Shigella dysenteriae*, chestnut bur, antibacterial activity, metabolomics, transcriptomics

## Abstract

*Shigella dysenteriae* is a highly pathogenic microorganism that can cause human bacillary dysentery by contaminating food and drinking water. This study investigated the antibacterial activity of chestnut bur polyphenol extract (CBPE) on *S. dysenteriae* and the underlying mechanism. The results showed that the minimum inhibitory concentration (MIC) of CBPE for *S. dysenteriae* was 0.4 mg/mL, and the minimum bactericidal concentration (MBC) was 1.6 mg/mL. CBPE treatment irreversibly disrupted cell morphology, decreased cell activity, and increased cell membrane permeability, cell membrane depolarization, and cell content leakage of *S. dysenteriae*, indicating that CBPE has obvious destructive effects on the cell membrane and cell wall of *S. dysenteriae*. Combined transcriptomic and metabolomics analysis revealed that CBPE inhibits *S. dysenteriae* by interfering with ABC protein transport, sulfur metabolism, purine metabolism, amino acid metabolism, glycerophospholipid metabolism, and some other pathways. These findings provide a theoretical basis for the prevention and treatment of *S. dysenteriae* infection with extract from chestnut burs.

## 1. Introduction

*Shigella dysenteriae* is a common cause of bacillary dysentery in humans and primates. Its infection is followed by typical symptoms characterized by abdominal pain, diarrhea, and fever [1]. As an important global public health problem, *S. dysenteriae* infection is particularly prominent in developing countries and is prevalent in the food industry, including meat and dairy products, as well as in markets and food factories [2]. After ingestion together with food and drinking water, *S. dysenteriae* can migrate through the small intestine to the colon after survival in the acidic environment of the stomach, and then be immersed into the colon mucosa and cause local inflammation or systemic infection. Various antibiotics, such as penicillin and tetracycline and its derivatives, are often used to fight against bacterial infections. However, the long-term and extensive use of these antibiotics has led to increases in bacterial resistance and significant decreases in their effectiveness. Moreover, the excessive use of antibiotics will also destroy normal intestinal flora, leading to an imbalance in intestinal flora and the induction of various diseases; therefore, the search for safe and effective antibacterial drugs has become a research hotspot [3].

Chestnut (*Castanea Mollissima* Blume) is a traditional nut in China with a long cultivation history, and its cultivation area, yield, and quality rank first in the world. By 2020, the planting area of chestnut in China had reached 1.8 million hectares, with an annual output of more than 1.9 million tons, which accounts for over 80% of the world’s total chestnut production [4]. Large amounts of by-products and wastes are generated in the harvest and processing of chestnuts, such as chestnut burs, shells, flowers, and leaves, which together account for about 60% of the total weight of chestnuts [5]. Due to their specific functional compounds, different chestnut by-products have potential applications in the pharmaceutical, cosmetic, food, and leather industries [6,7]. Chestnut burs, which account for about 20% of the total weight of chestnuts, are usually burned after harvest to reduce the propagation of insect larvae [8]. The processing and reuse of these solid residues have become a priority, with the aim to reduce the environmental and economic impact of agro-industrial processes and obtain new functional products. Chestnut burs are low in protein and fat but have abundant polyphenols, such as vescalagin/castalagin, phenolic acids (gallic, ellagic, protocatechuic, cholorgenic acid), and flavonoids (apigenin, quercetin, and quercetin 3-*O*-β-glucoside) [9,10]. It has been reported that polyphenols and their polyphenol-rich extracts have a broad spectrum of antibacterial effects on human pathogens, particularly some phenolic compounds such as quercetin ferulate, rutin, and catechins, which have become a research hotspot in recent years [11,12,13]. Previous research has found that chestnut bur extract has inhibitory effects on the mycelial growth and spore germination of *Alternaria alternata* and *Fusarium solani* [14], and its polyphenol components mainly comprise ellagitannin and flavonol [15]. Silva et al. reported that the alcoholic extract of chestnut burs is effective against *Staphylococcus aureus* (*S. aureus*) [16]. Fernández-Agulló demonstrated that the water extract of chestnut burs has an inhibitory effect on *S. aureus* [9]. Moreover, there are very significant correlations between the antibacterial activity and oxygen free radical scavenging ability of different varieties of chestnut bur extract [17]. These studies have indicated that the extract of chestnut burs is a potential bactericide with strong bactericidal activity.

However, there have only been a few studies on the antibacterial activity of chestnut bur extract against *S. dysenteriae*, and the action mechanism remains unclear. Here, we studied the bactericidal activity of chestnut bur polyphenol extract (CBPE) against *S. dysenteriae* and further investigated the mechanism underlying its effect on *S. dysenteriae* from the perspectives of physiology, transcriptome, and metabolome. Elucidation of the molecular mechanism is expected to provide a theoretical basis for the prevention and control of bacillary dysentery and relevant food-borne diseases and promote the functional component-oriented utilization of chestnut waste.

## 2. Results

### 2.1. CBPE Components

**Figure 1:**
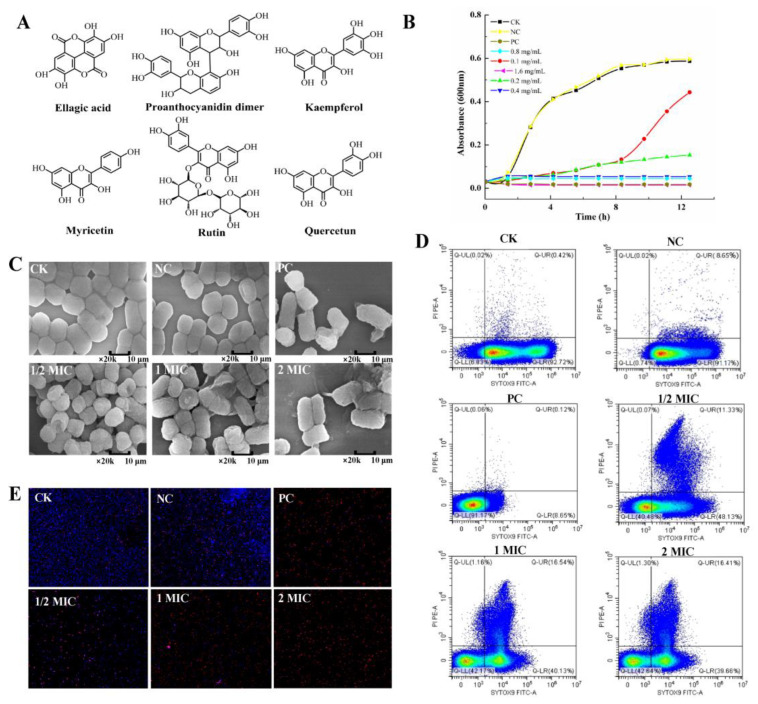
Bactericidal activity of CBPE against *S. dysenteriae*. Chemical structure of CBPE (**A**). Effects of CBPE on the growth curve of *S. dysenteriae* (**B**). SEM observation of *S. dysenteriae* using CMBE (**C**). Fluorescence spot images of *S. dysenteria* treated with CBPE were analyzed with flow cytometry. Events in different quadrants correspond to different populations: dead cells (Q-UL), membrane-damaged intermediate populations (Q-UR), living cells (Q-LR), and weakly stained cells (Q-LL), with the numbers in each quadrant representing the relative percentage of each population (**D**). The effect of CBPE on bacterial viability was observed using a confocal laser. DAPI-stained cells are labeled blue and PI-stained cells are labeled red (**E**). CK: blank group; NC: negative control group; PC: positive control group; 1/2MIC, MIC, and 2MIC indicate CBPE treatment groups at 1/2MIC, MIC, and 2MIC concentrations, respectively.

CBPE components were qualitatively and quantitatively analyzed using HPLC−ESI−MS/MS. The calibration curve for each compound was prepared by measuring standard solutions with six concentrations (0.05, 0.2, 1.0, 5.0, 10.0, and 50.0 mg/L). As shown in Figure 1A, six major polyphenols were identified in CBPE, including ellagitannin (155.1 mg/g), proanthocyanidin dimer (1.3 mg/g), kaempferol (2.2 mg/g), myricetin (0.5 mg/g), rutin (0.1 mg/g), and quercetin (0.3 mg/g).

### 2.2. Antibacterial Activity of CBPE against S. dysenteriae

As shown in Figure 1B, CBPE treatment completely inhibited the growth of *S. dysenteriae* during the logarithmic growth phase, with MIC of 0.4 mg/mL (MIC_50_, 0.14 mg/mL) and MBC of 1.6 mg/mL. The SEM results showed that *S. dysenteriae* in the CK group had a full and round morphology as well as complete cell walls and membrane structures (Figure 1C). After 6 h of treatment, CBPE at 1/2MIC resulted in the shrinking of bacterial cells, and that at MIC led to the destruction of the cell structure. Treatment with CBPE at 2MIC destroyed the cell membrane and led to the dissolution and leakage of internal components. These results demonstrated that CBPE could disrupt the normal morphology of *S. dysenteriae* in a concentration-dependent manner.

The flow scatter plot in Figure 1D shows the changes in bacterial activity induced with CBPE treatment. The proportion of living cells in all bacteria without CBPE treatment was about 92.72%. However, treatment with CBPE at different concentrations (1/2MIC, MIC, and 2MIC) decreased the proportion of viable bacteria, which was 48.13%, 40.13%, and 39.66%, respectively. After exposure to CBPE at 1/2MIC and MIC, the proportion of damaged cells in the Q-UR region was 11.33% and 16.54%, respectively, indicating the destruction of some cell membranes of *S. dysenteriae*. The CLSM results showed that more than 95% of CK group bacteria emitted blue fluorescence (Figure 1E), indicating their high vitality and intact cell membranes. With increasing CBPE concentration, the number of viable bacteria decreased significantly, accompanied by an increase in red fluorescence and a decrease in blue fluorescence, indicating a significant increase in the number of bacteria with disrupted cell membranes. These results are consistent with those of flow cytometry.

### 2.3. Effect of CBPE on the Cell Membrane and Cell Wall of S. dysenteriae

#### 2.3.1. Effect of CBPE on the Cell Membrane Permeability of *S. dysenteriae*

**Figure 2:**
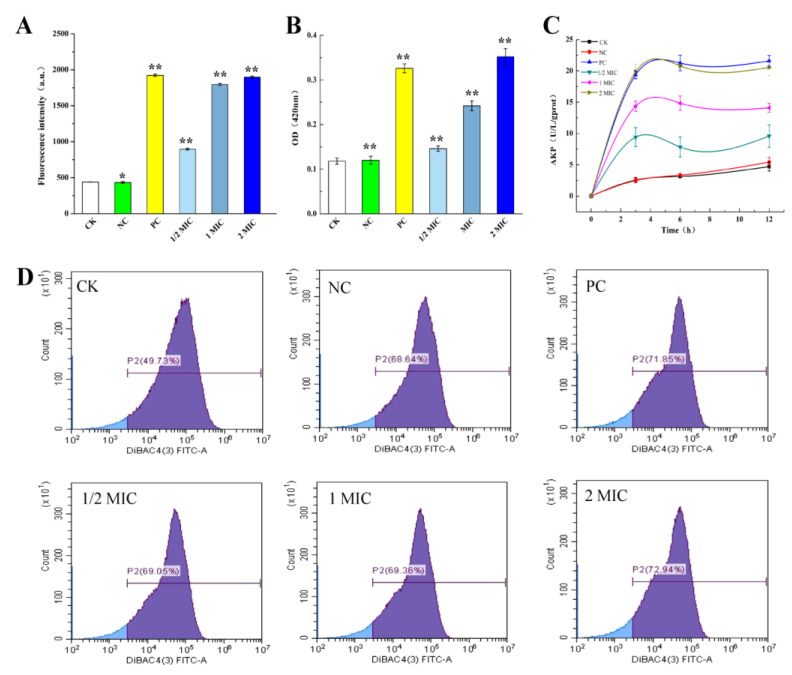
Effects of CBPE on the somatic membrane and cell wall of *S. dysenteriae*. Influence on the permeability of the outer cell membrane (**A**). Effect on cell intimal permeability (**B**). Effect on alkaline phosphatase activity (**C**). Effect on cell membrane potential (**D**). Data are expressed as mean ± SD (*n* = 3). ** *p* <0.01, * *p* < 0.05 vs. CK.

The effect of CBPE on the outer membrane permeability of *S. dysenteriae* was determined using fluorescence probe NPN. As shown in Figure 2A, the fluorescence intensity increased with increasing CBPE concentration. This phenomenon may be ascribed to the increase in outer membrane permeability, which led to the entry of NPN into the hydrophobic environment inside the cell and thereby increased the fluorescence intensity. As shown in Figure 2B, the addition of CBPE significantly increased the OD_420nm_ value of the *S. dysenteriae* suspension. When the concentration of CBPE was increased from 1/2MIC to 2MIC, the OD_420nm_ value of the bacterial suspension showed a dramatic increase, indicating that CBPE induces a significant increase in the inner membrane permeability of *S. dysenteriae* in a concentration-dependent manner.

#### 2.3.2. Effect of CBPE on the Cell Wall Integrity of *S. dysenteriae*

AKP is located between the cell wall and the membrane, and measurement of AKP can generally reflect the integrity of the bacterial cell wall [18]. As shown in Figure 2C, CBPE treatment resulted in significantly higher extracellular AKP content of *S. dysenteriae* than CK. The AKP concentration increased significantly within 3 h, and AKP leakage reached a maximum of 22 U/L/gprot at 5 h, which was positively correlated with CBPE concentration, indicating that CBPE has a certain destructive effect on the cell wall.

#### 2.3.3. Effect of CBPE on the Cell Membrane Potential of *S. dysenteriae*

As shown in Figure 2D, CBPE treatment destroyed the potential balance between the inner and outer membrane of cells, resulting in the rapid entry of DiBAC4(3) into the membrane under the action of positive potential and an increase in intracellular fluorescence intensity from 49.73% to 72.94%. These results indicated that CBPE treatment depolarizes the cell membrane of *S. dysenteriae*, and the decrease in stability of the membrane potential affects the membrane permeability, transmembrane transport, and other activities, resulting in the failure of normal bacterial growth.

### 2.4. Effects of CBPE on Cell Contents

#### 2.4.1. Effect of CBPE on the Cell Membrane Potential of *S. dysenteriae*

**Figure 3:**
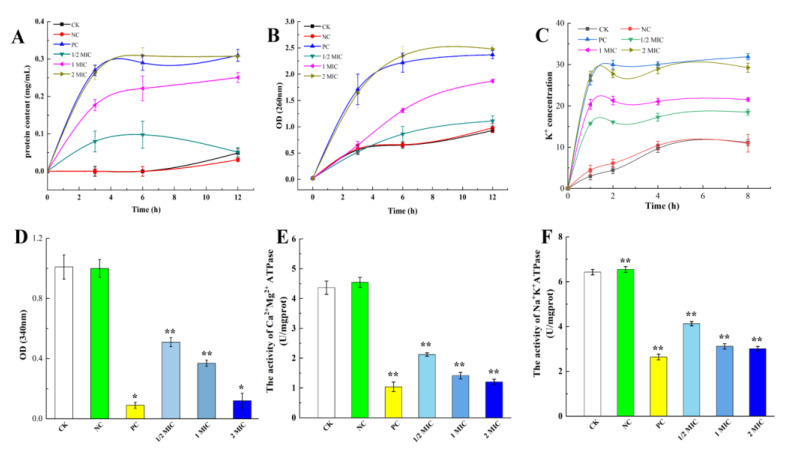
Leakage of protein, macromolecule, K^+^, ATP and ATPase from *S. dysenteriae* treated with CBPE. Protein leakage (**A**); macromolecule leakage (**B**); cell K^+^ leakage (**C**); intracellular ATP content (**D**); Ca^2+^ Mg^2+^-ATPase (**E**); and Na^+^ K^+^-ATPase activities (**F**). Data are expressed as mean ± SD (*n* = 3). ** *p* < 0.01, * *p* < 0.05 vs. CK.

As shown in Figure 3A, the protein concentration in the CK group exhibited no significant change with time. The extracellular protein concentration of *S. dysenteriae* treated with CBPE at 1/2MIC and MIC increased with the extension in treatment time, and the leakage at 12 h was 0.051 ± 0.013 mg/mL and 0.251 ± 0.013 mg/mL, respectively. After 2MIC treatment, the extracellular protein concentration showed a linear increase in the first 4 h, and then the protein release rate slowed down and gradually flattened, reaching a maximum value of 0.309 ± 0.021 mg/mL at 6 h. These results indicated that the protein leakage of *S. dysenteriae* is positively correlated with the concentration of CBPE.

#### 2.4.2. Effect of CBPE on the Macromolecular Leakage of *S. dysenteriae*

As shown in Figure 3B, the amount of macromolecular leakage in the CBPE treatment group was significantly higher than that in the CK group, and it increased with the extension in treatment time, indicating that CBPE could promote membrane permeability and result in the leakage of macromolecular substances. The concentrations of macromolecules in suspensions treated with CBPE at 1/2MIC, MIC, and 2MIC increased by 1.19-, 2.01-, and 2.67-fold compared with that of the CK, respectively, indicating that CBPE acts on the cell membrane in a concentration-dependent manner.

#### 2.4.3. Effect of CBPE on the Extracellular K^+^ Content of *S. dysenteriae*

As shown in Figure 3C, after 1 h of treatment with CBPE, the extracellular K^+^ concentration of *S. dysenteriae* increased rapidly, and the leakage of K^+^ in the 2MIC group reached 29.25 g/L. Subsequently, the increase in K^+^ leakage in the CBPE treatment group slowed down and was not significant after 2 h.

#### 2.4.4. Effects of CBPE on the ATP Content and ATPase Activity in *S. dysenteriae* Cells

As shown in Figure 3D, compared with that of the CK group, the intracellular ATP content of *S. dysenteriae* in the CBPE (1/2MIC, MIC, and 2MIC) treatment groups decreased by 49.50%, 63.37%, and 88.12%, respectively. These results demonstrated that CBPE significantly inhibits the intracellular ATP content of *S. dysenteriae* in a concentration-dependent manner (*p* ≤ 0.05). Figure 3E,F shows the effects of CBPE on the activity of two typical ATPase enzymes in *S. dysenteriae* cells. Under CBPE treatment at MIC and 2MIC, the activities of the two ATPase enzymes in *S. dysenteriae* cells significantly decreased (*p* < 0.01), indicating that CBPE has a significant inhibitory effect on the two ATPase enzymes in *S. dysenteriae*.

### 2.5. Transcriptomics Analysis

**Figure 4:**
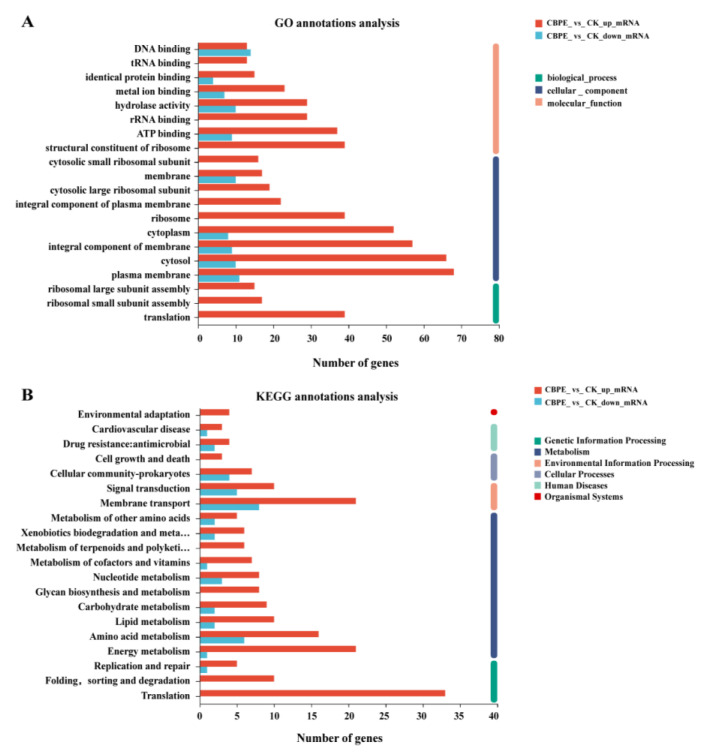
Bioinformatics analysis of differentially expressed genes (DEGs). Enriched gene ontology (GO) terms of DEGs between control and 1/2MIC CBPE treatment groups (**A**). Scatter diagram showing the top 20 KEGG enrichment pathways for DEGs (**B**).

As a result of the transcriptomic analysis, 3117, 3191, 3339, and 3245 unigenes were found under the CK, PC, NC, and CBPE treatments, respectively (Figure S1). A total of 357 DEGs (257 up-regulated and 100 down-regulated) were screened in the CBPE treatment group (relative to the CK) (Table S1). The GO enrichment analysis (Figure 4A) showed that these DEGs could be divided into three categories: cellular component (CC), biological process (BP), and molecular function (MF) [19]. Classification of the CC showed that the DEGs were mainly distributed in the cell membrane, an integral component of the membrane, cytosol, and cytoplasm. Classification of BP demonstrated that the DEGs were mainly involved in metabolic processes, cellular processes, and cell localization. The MF classification results showed that the main molecular functions of DEGs were associated with the metabolism of the cell plasma membrane, ribosomes, ATP synthesis, cytosol, and translation. KEGG enrichment analysis showed that CBPE treatment led to differential expression of genes related to membrane transport, translation, energy metabolism, amino acid metabolism, and lipid metabolism (Figure 4B).

### 2.6. Metabolomics Analysis

**Figure 5:**
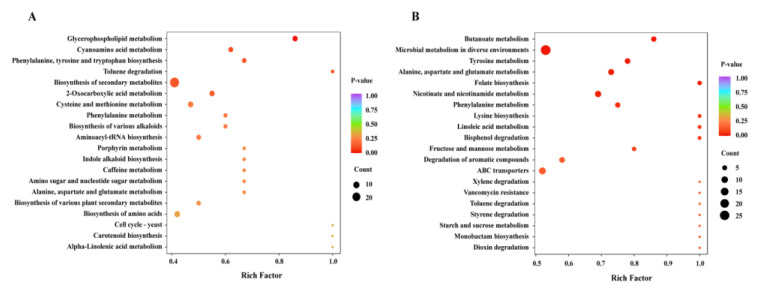
Metabolic profile analysis of *S. dysenteriae* in the control (CK) and 1/2MIC CBPE treatment groups. KEGG enrichment analysis of DMs in the positive ion mode (**A**) and in the negative ion mode (**B**).

In the metabolomics analysis, a total of 3286 metabolites were detected, with 1873 and 1413 metabolites being detected in the positive and negative ion modes, respectively (Table S2 and Table S3). PCA of the metabolites showed that after CBPE treatment, the scattered points corresponding to the metabolite samples of *S. dysenteriae* were relatively clustered (Figure S2), indicating good repeatability within the group. A total of 678 (395 positive and 283 negative) differential metabolites (DMs) were found between CBPE and CK treatments (Tables S2 and S3), which mainly involved amino acids, benzene and its derivatives, alcohols, amines, and glycerol phospholipids. The KEGG enrichment analysis (Figure 5A,B) showed that most of the DMs in the positive ion mode were involved in secondary metabolites, glycerophospholipid metabolism, aromatic amino acid metabolism, amino acid metabolism, and some other pathways. The DMs in the negative ion mode were mainly involved in microbial metabolism in diverse environments, alanine, aspartate and glutamate metabolism, nicotinate and nicotinamide metabolism, tyrosine metabolism, ABC transporters, and some other pathways.

### 2.7. Correlation Analysis of Metabolism and Transcription

**Figure 6:**
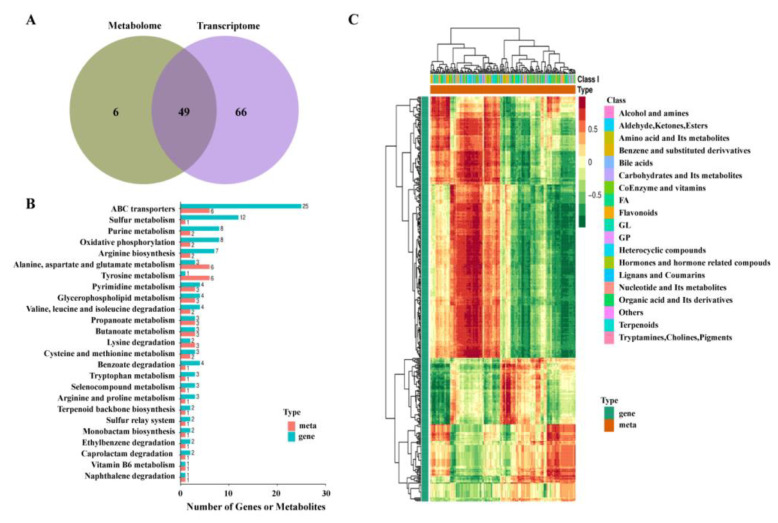
Integrated analysis of transcriptomic and metabolomic results of *S. dysenteriae* treated with 1/2MIC CBPE. Comparison of the number of pathways covered by DEGs and DMs (**A**); Ten of the most enriched pathways co-engaged by DEGs and DMs (**B**). Spearman correlation heatmap of DEGs and DMs (**C**).

In order to further analyze the action pathway of CBPE on *S. dysenteriae*, the RNA data obtained from the transcriptome and metabolome were normalized and statistically analyzed. By consensus, 49 metabolic pathways involved in both transcriptomics and metabolomics were identified (Figure 6A), and the most important pathways for differential enrichment included ABC protein transport, sulfur metabolism, purine metabolism, arginine metabolism, tyrosine metabolism, and glycerolipid metabolism (Figure 6B). To directly reveal the difference in expression patterns of DEGs and DMs, a correlation hierarchical cluster analysis was performed (Figure 6C), and a high Spearman correlation coefficient was obtained, indicating that the results of transcriptome and metabolome were significantly correlated.

## 3. Discussion

At present, the main treatment of bacillary dysentery is the use of antibiotics, but long-term use of antibiotics can cause the generation of drug-resistant pathogens [20], and drug-resistant strains have been isolated from various food sources [21]. Therefore, there is an urgent need to find new antimicrobial agents against *S. dysenteriae*. Previously, several studies revealed the antibacterial effects of chestnut bur extract on a variety of foodborne pathogens, such as *Staphylococcus aureus*, *E. coli, L*. monocytogenes, and *Botrytis cinerea* [14,15,16,17]. Our results showed that the MIC and MBC of CBPE on *S. dysenteriae* are 0.4 mg/mL and 1.6 mg/mL, respectively. These values are slightly lower than the MIC (2 mg/mL) and MBC (4 mg/mL) of *Shigella flexneri* reported by Kang et al. [22], indicating a better antibacterial effect of CBPE on *S. dysenteriae*. To further analyze the antibacterial activity of CBPE on *S. dysenteriae*, growth curves of *S. dysenteriae* treated with different concentrations of CBPE were plotted, which further confirmed that CBPE has a concentration-dependent antibacterial effect on *S. dysenteriae*.

The cell wall is the first line of defense for bacterial cells to resist the killing ability of antibacterial substances [23]. When the cell wall integrity of bacteria is destroyed, the cell wall will fail to play its role as an osmotic barrier, which will cause the leakage of intracellular components. AKP exists between the cell wall and cell membrane and is generally not secreted outside the cell. However, with damage to cell walls, AKP leaks into the extracellular environment and increases the AKP activity in the extracellular environment [24]. Therefore, cell wall integrity can be determined by detecting the AKP activity in the extracellular environment. In this study, we found that CBPE treatment can destroy the cell wall integrity of *S. dysenteriae* within 3 h. Compared with the effect of cinnamon-leaf essential oil reported by Huang et al., which increases the AKP activity in the extracellular environment of *S. dysenteriae* within 5 h [25], CBPE seems to have a more efficient effect on the AKP activity of *S. dysenteriae*.

Bacteria absorb nutrients from the external environment through osmosis and selective absorption of cell membranes to maintain normal physiological functions. When the osmotic balance in the cell membrane is broken, bacterial growth and metabolism will be inhibited [26]. In this study, with increasing CBPE concentration, the permeability of the inner and outer membranes of *S. dysenteriae* cells was gradually enhanced. Cell membrane potential is also an important indicator of bacterial physiological metabolism. Flow cytometry with membrane potential staining showed that CBPE triggered a decline in cell viability of *S. dysenteriae* by disrupting and depolarizing the cell membrane. Kang et al. found that ferulic acid can cause irreversible changes in the permeability of the cell membrane of *Shigella flexneri*, resulting in the loss of the cell’s ability to maintain the membrane potential and intracellular macromolecules [22]. Therefore, we speculate that CBPE treatment disrupts the cell membrane and depolarizes the membrane potential of *S. dysenteriae*, breaking the internal and external permeability balance in the cell membrane and causing the leakage of cell contents. This speculation can be confirmed by measuring the content of macromolecules such as proteins and DNA in extracellular inclusions. Leakage of intracellular lysates such as protein and K^+^ is an important indicator of membrane damage and loss of membrane integrity [27]. After CBPE treatment at different concentrations, the cell contents (protein and K^+^) of *S. dysenteriae* showed varying degrees of leakage, which is consistent with the detection results of flow cytometry, further indicating that CBPE can increase the permeability of bacterial cell membranes and lead to the leakage of intracellular K^+^, nucleic acid, and protein. In addition, the SEM and CLEM results also support the results of the cell wall integrity determination, flow cytometry analysis, and protein and K^+^ leakage analysis. The inhibitory pattern of CBPE on *S. dysenteriae* may be related to the destruction of the cell wall and membrane structure, resulting in changes in cell morphology, loss of cell viability, and then death of *S. dysenteriae*.

ATP is composed of adenine, ribose, and three phosphoric acid molecules and plays a key role in physiological processes such as energy supply, signaling, and biosynthesis in bacteria [28]. A reduction in ATP content will also affect the synthesis of macromolecular substances such as proteins and nucleic acids as well as the physiological metabolism of bacteria. ATPase is a membrane marker enzyme and an important ion exchanger. Ion pumps in the cell membrane, including Na^+^/K^+^-ATPase, Mg^2+^-ATPase, and Ca^2+^-ATPase, are embedded between the phospholipid bilayer and have functions including maintaining cell osmotic pressure balance, membrane resting potential, and information conduction [18]. Our results showed that CBPE could significantly inhibit the ATP content and ATPase activity of *S. dysenteriae*. It is possible that the antibacterial components in CBPE increase the permeability of the cell membrane of the tested bacteria and cause the leakage of intracellular substances, and, at the same time, the antibacterial substances of small- and medium-sized molecules in the extract can also pass through the cell membrane and then combine with intracellular substances, thereby disrupting the normal energy metabolism of *S. dysenteriae* and further affecting the content of ATP and the activity of ATPase.

Transcriptomic and metabolomic analyses further demonstrated that many ATP-related genes and small-molecular metabolites were affected by CBPE treatment, especially those related to ABC transporters and energy metabolic pathways. ABC transporters play a key role in the transmembrane transport of molecules, mainly binding with ATP to transport sugars, amino acids, and proteins across biofilms [29]. In addition, ABC transporters are involved in the tolerance and resistance of cells to toxic substances that assist their survival in an unfavorable environment [30]. The differential expression of ABC transporter-related genes and metabolites suggested that CBPE affects metabolite transport in *S. dysenteriae* and prevents the efflorescent system from pumping antibacterial components of CBPE out of the cell. Moreover, due to its lipophilicity, CBPE may be directly incorporated into the membrane lipid bilayer, which will reduce the fluidity of the cell membrane, thereby inhibiting the membrane transport of amino acids through specific transmembrane integrins.

Another interesting finding of this study is that CBPE significantly disrupts glycerol phospholipid metabolism (choline, acetylcholine, phosphatidylcholine (PC), lysophosphatidylcholine (LPC)) in *S. dysenteriae*. Choline and acetylcholine are components of glycerophospholipid metabolism, playing an important role in the formation and maintenance of cell structure, material permeability, and cell information transmission [31]. PC is the main phospholipid in the plasma membrane, as well as a downstream product of choline, and plays an important role in the integrity and fluidity of the membrane [32]. Equally important, glycerophospholipids can help bacteria form stable biofilm structures and build a fat barrier between the cell interior and the surrounding environment [33]. Taking the above results together, it can be inferred that CBPE treatment increases cell membrane permeability and intracellular solute leakage of *S. dysenteriae*, leading to an imbalance in intracellular osmotic pressure. It has been reported that many antibacterial substances, such as polyphenols, can increase bacterial membrane permeability and inhibit bacterial activities by affecting the expression of osmoprotectant-related genes and the content of organic solutes, which can ultimately lead to cell inactivation [34,35]. Our physiological study revealed that CBPE could destroy the morphology of the cell membrane and cell wall of *S. dysenteriae*, and significantly inhibit the metabolic activity of the tested bacteria, which may be related to the disorder of glycerol phospholipid metabolism.

## 4. Materials and Methods

### 4.1. Reagents and Strains

*S. dysenteriae* (BNCC 103609) was purchased from North Na Biotechnology Co., Ltd., (Hebei, China). 2-nitrophenyl-P-D-galactoside pyranoside (ONPG), N-phenyl-1-naphthylamine (NPN), Bis-(1,3-dibutylbarbituric acid) trimethine oxonol (DiBAC4(3)), propidium iodide (PI), and 4′,6-diamidino-2-phenylindole (DAPI) were purchased from Solaibao Technology Co., Ltd., (Beijing, China).The reagents used in the experiment are all of analytical grade.

### 4.2. Plant Materials and Extraction

Chestnut burs were collected from Qinglong County, Hebei Province, in October 2021 (longitude: 118.95, latitude: 40.40). After drying, the samples were crushed and screened with 40 mesh for further use. After petroleum ether degreasing and drying, chestnut bur powder was extracted with 45% ethanol (liquid to material ratio 20:1) at 50 °C for 2 h. The extraction solution was centrifuged at 7500 r/min for 10 min, and the supernatant was collected and concentrated under reduced pressure. Subsequently, the concentrated solution was extracted with ethyl acetate and freeze-dried (Alpha 2–4 Dplus, Marin Christ, Osterode, Germany).

### 4.3. Analysis of CBPE

The CBPE components were analyzed using high-performance liquid chromatography-mass spectrometry (Triple TOF 5600+, AB SCIEX, Concord, ON, Canada). In the negative ion mode, the mobile phase was 0.1% CH_3_COOH-H_2_O (solvent A) and acetonitrile (solvent B). The gradient elution conditions were 100% A (0 min), 50% A (10 min), 5% A (13 min), and 100% B (15 min). The flow rate was 0.3 mL/min. Double online detection was carried out with a PDA using 280 nm and 320 nm. Spectra were recorded in negative ion mode between m/z 100 and 2000. The compounds were characterized according to their UV, mass spectra, and retention time and by comparison with authentic standards when available. Purchased standards (purity > 98%) were used for quantitative analysis of the CBPE components. The reference compound was selected according to the principle of structure-related target standard (chemical structure or functional group). The calibration curve for ellagic acid was used to quantify ellagitannin. The calibration curve for procyanidin B2 was used to quantify proanthocyanidin dimer. Kaempferol, myricetin, rutin, and quercetin were quantified with their own standards.

### 4.4. Determination of CBPE Antibacterial Activity 

#### 4.4.1. Determination of the Minimum Inhibitory Concentration (MIC) and Maximum Bactericidal Concentration (MBC)

CBPE was configured as a solution with a concentration of 3.2 mg/mL (1.5% DMSO as co-solvent). By half dilution, a certain volume of CBPE solution was mixed with Luria-Bertani (LB) medium, so that the concentration of CBPE was set as 1.6, 0.8, 0.4, 0.2, and 0.1 mg/mL. The mixture was then poured into a Petri dish. After cooling and solidification, the mixture was evenly coated with bacterial suspension and cultured at 37 °C for 24 h. MIC is the lowest concentration of CBPE at which bacterial growth was completely inhibited, and MBC is the lowest concentration at which CBPE showed bactericidal activity (inhibiting 99.9% of the tested bacteria). MIC_50_ values were calculated by regressing the growth inhibition percentage against the log-transformed CBPE concentration. Sterile water was used as the blank control (CK); 1.5% DMSO was used as the negative control (NC); and ciprofloxacin was used as the positive control (PC).

#### 4.4.2. Growth Curve Assay

CBPE was mixed with LB medium to obtain final concentrations of 1.6, 0.8, 0.4, 0.2, and 0.1 mg/mL, respectively. *S. dysenteriae* was inoculated into each LB medium. About 200 μL of the mixture was placed in a 96-well plate, and the absorbance at 600 nm was measured every 1 h for 12 h.

### 4.5. Inhibition Mechanism of CBPE

#### 4.5.1. Observation of *S. dysenteriae* Morphology by Scanning Electron Microscope (SEM)

First, 1 × 10^6^ CFU/mL *S. dysenteriae* was mixed with the sample solution (1/2MIC, MIC, and 2MIC) and cultured with 180 r/min shock at 37 °C. The same amount of mixture was placed into the centrifugal tube at the treatment time of 6 h, cleaned three times with PBS buffer solution, and fixed with 2.5% pentanediol, followed by gradient dehydration with 30–100% ethanol aqueous solution in turn. After drying, gold was sprayed and observed wit SEM (S-3400 N, Hitachi, Tokyo, Japan).

#### 4.5.2. Observation of Bacterial Activity using Confocal Laser Scanning Microscope (CLSM)

*S. dysenteriae* bacterial solution was mixed with CBPE solution for 6 h. Then, 1.5 mL of the mixture was placed in a centrifuge tube, washed three times with PBS buffer solution, and then suspended in 1 mL PBS. Subsequently, PI and DAPI were stained in a dark environment for 15 min and observed under CLSM (FV 3000, Olympus, Tokyo, Japan).

#### 4.5.3. Cell Viability Assay

*S. dysenteriae* suspension was added with CBPE (1/2MIC MIC 2MIC) solution and cultured at 37 °C for 6 h. The mixture was then centrifuged at 8000 r/min for 10 min, washed with sterile PBS for three times, added with SYTO9 and PI, and cultured in the dark for 15 min. After centrifugation at 8000 r/min for 10 min, the bacteria were collected and detected using flow cytometry.

#### 4.5.4. Analysis of Outer/Inner Membrane Permeability

According to the method of Johnson et al. [36], the samples were added into the bacterial suspension until the final concentration was 1/2MIC, MIC, and 2MIC, respectively, and mixed with 20 μL NPN (1 mmol/L) fluorescent agent at 37 °C for 1 h. The fluorescence intensity was detected with a fluorescence photometer (F-7000, Hitachi, Tokyo, Japan). The excitation wavelength was 350 nm, and the emission wavelength was 430 nm. According to the method of Huang et al. [37], *S. dysenteriae* was treated with CBPE for 2 h. After centrifugation at 8000 r/min for 10 min, the supernatant was collected and added to ONPG until the final concentration was 30 mM. After incubation at 37 °C for 30 min, the absorbance at 420 nm was determined.

#### 4.5.5. Detection of Cell Membrane Potential

The fluorescent dye DiBAC4(3) was added into the bacterial suspension and incubated at 37 °C away from light for 15 min. The final concentrations of the samples were 1/2MIC, MIC, and 2MIC, respectively. The effect of CBPE treatment on the cell membrane potential of *S. dysenteriae* was analyzed using flow cytometry.

#### 4.5.6. Cell Wall Integrity Assay

First, 1 × 10^6^ CFU/mL bacterial suspension was mixed with CBPE (1/2MIC, MIC, and 2MIC), and 1.5 mL bacterial solution was taken at 3, 6, and 12 h, respectively. Alkaline phosphatase (AKP) activity was detected according to the instructions of the AKP detection kit (Solaibao Technology Co., Ltd., Beijing, China).

#### 4.5.7. Assay of Macromolecular Substances and Protein Leakage

*S. dysenteriae* bacterial suspension was mixed with CBPE solution. About 1.5 mL bacterial solution was taken at 3, 6, and 12 h, respectively, and the absorbance of macromolecular substances in the supernatant was measured at 260 nm. The protein content in the supernatant was determined according to the instructions of the Bradford protein concentration assay kit (Jiancheng Bioengineering Institute, Nanjing, China).

#### 4.5.8. Determination of K^+^ Content

*S. dysenteriae* bacterial suspension was mixed with CBPE (1/2MIC, MIC, and 2MIC) solution. The same amount of bacterial liquid was taken at 1, 2, 4, and 8 h, respectively. The content of K^+^ was measured with an atomic absorption spectrometer (Purkinje General Instrument Co., Ltd., Beijing, China).

#### 4.5.9. Determination of ATP Content and ATPase Activity

Intracellular ATP of *S. dysenteriae* was collected according to the method of Kang et al. [18]. The ATP content was determined using an ATP kit (Solaibao Technology Co., Ltd., Beijing, China). The enzyme adenosine triphosphate was determined using an ATPase assay kit (Jiancheng Bioengineering Institute, Nanjing, China).

### 4.6. Transcriptomic Analysis 

Total RNA was extracted from the tissue using TRIzol^®^ Reagent according to the manufacturer’s instructions (Invitrogen, Waltham, MA, USA) and genomic DNA was removed using DNase I (TaKara, Kusatsu, Japan). RNA libraries were constructed using an Illumina (San Diego, CA, USA) TruSeqTM RNA sample preparation kit. The data generated with the Illumina platform were used for bioinformatics analysis. All analyses were performed using Shanghai Megbio’s I-Sanger cloud platform (www.i-sanger.com, accessed on 19 October 2022). Differential expression analyses were performed using edgeR (http://www.bioconductor.org/packages/2.12/bioc/html/edgeR.html, accessed on 10 September 2023), DESeq2 (http://bioconductor.org/packages/release/bioc/html/DESeq2.html, accessed on 10 September 2023), and DESeq (http://www.bioconductor.org/packages/release/bioc/html/DESeq.html, accessed on 10 September 2023). The software Goatools (version 1.3.1) was used for enrichment analysis, and four multiple test methods (Bonferroni, Holm, Sidak, and false discovery rate) were used to correct the *p*-value to control the false detection rate. |log2 (fold change)| > 1 and genes with *p* < 0.05 (where the *p*-value is the value after correction) were considered as differentially expressed genes (DEGs). The Kyoto Encyclopedia of Genes and Genome (KEGG) was enriched and analyzed with KOBAS 2.0 (http://kobas.cbi.pku.edu.cn/home.do, accessed on 10 September 2023) to visually identify DEGs in the KEGG pathway.

### 4.7. Non-Targeted Metabolomics

#### 4.7.1. Metabolome Sample Processing

*S. dysenteriae* was treated with 1/2MIC CBPE for 6 h. The bacteria were collected and washed with sterile water three times. Lysis buffer (80% methanol) was added to the 100 μg sample ground with liquid nitrogen. The mixture was mixed in a vortex, treated with a tissue crusher at 45 Hz for 4 min, and ultrasound for 5 min (ice water bath). Then, the sample was allowed to stand at –20 °C for 30 min. Subsequently, 200 μL supernatant was mixed with 5 μL internal standard (1 mg/mL of dichlorophenylalanine) and transferred to the LC-MS sample bottle after centrifugation (4 °C, 14,000× *g*, 15 min).

#### 4.7.2. UPLC-MS Analysis

The metabolites were analyzed using Agilent 1290 II (Agilent, Santa Clara, CA, USA) with ACQUITY UPLC HSS T3 column (1.8 µm, 2.1 × 100 mm) with mobile phase A: ultra-pure water (0.1% formic acid) and mobile phase B: acetonitrile (0.1% formic acid). The elution gradient was set as: 0 min, 95% A; 0–11 min, 95–10% A; 11–12 min, 10% A; 12–12.1 min, 10–95% A; and 12.1–14 min, 95% A. The flow rate was 0.4 mL/min.

MS was performed using QTOF/MS-6545 (Agilent, Santa Clara, CA, USA). All analyses were performed in electrospray ionization (ESI±) mode under the following conditions: The electrospray voltage was 2.5 kV (positive ion mode) and 1.5 kV (negative ion mode); the heater temperature was 325 °C and the capillary temperature was 350 °C; the sheath gas flow rate was 45 arb; and the auxiliary gas flow rate was 8 arb.

#### 4.7.3. Data Processing

Proteo Wizard (http://proteowizard.sourceforge.net/, accessed on 10 September 2023) was used to convert the original mass spectrometry data (.wiff) into mzXML format, and the XCMS program was used to correct retention time, peak alignment, peak extraction, peak integration, and peak identification. After calibration and screening, the metabolites were identified by searching our laboratory’s self-established database, integrated public database, and AI prediction database and using the metDNA method.

#### 4.7.4. Bioinformatics Analysis

Metabolome data were imported into SIMCA software (15.0 version) for principal component analysis (PCA), orthogonal partial least squares discriminant analysis (OPLS-DA), and OPLS-DA replacement test. Metaboanalyst (version 3.0) visualization software online (http://www.metaboanalyst.ca, accessed on 13 December 2022) was used for hierarchical cluster analysis and the analysis of metabolic pathways. The KEGG database (https://www.kegg.jp/kegg/, accessed on 13 December 2022) and Pub Chem database (https://pubchem.ncbi.nlm.nih.gov, accessed on 13 December 2022) were used to conduct qualitative analysis and search metabolites in the biosynthetic pathway.

### 4.8. Statistical Analysis

SPSS 20.0 version (SPSS Inc., Chicago, IL, USA) was used to perform one-way analysis of variance (ANOVA) and Tukey tests. Statistically, *p* < 0.05 indicated a significant difference.

## 5. Conclusions

In general, the main mechanism underlying the action of CBPE on *S. dysenteriae* can be summarized as follows. CBPE passes through the cell wall and combines with the cell membrane of *S. dysenteriae*, resulting in an increase in membrane permeability, imbalance of intracellular osmotic pressure, destruction of the electron transport chain, interference with cell energy metabolism, and a decrease in bacterial vitality or even bacterial death. In addition, CBPE also affects intracellular metabolic pathways such as ABC protein transport, sulfur metabolism, purine metabolism, arginine metabolism, tyrosine metabolism, and glycerolipid metabolism, suggesting that CBPE inhibits the growth of *S. dysenteriae* through multiple pathways. In summary, CBPE is a potent natural antibacterial candidate for *S. dysenteriae*. Further studies can be focused on the in vivo prevention of *S. dysenteriae* infection and invasion by CBPE and its application in the food industry.

## Data Availability

Data presented in this article are available at request from the corresponding author.

## Supplementary Materials

The following supporting information can be downloaded at: https://www.mdpi.com/article/10.3390/molecules28196990/s1, Figure S1: Venn diagram; Figure S2: PCA score plots; Figure S3: The effect of CBPE on the inhibition of *S. dysenteriae*; Table S1: DEGs between CK and 1/2 MIC CBPE treatment group; Table S2: DMs CK and 1/2 MIC CBPE treatment group in the positive ion mode; Table S3: DMs CK and 1/2 MIC CBPE treatment group in the negative ion mode.

## Sample Availability

Samples of the compounds are available from the corresponding authors.
