# Supplementary figures and images for: Effect of Chestnut (Castanea Mollissima Blume) Bur Polyphenol Extract on Shigella dysenteriae: Antibacterial Activity and the Mechanism
Source: Molecules. 2023 Oct 9;28(19):6990. doi: 10.3390/molecules28196990 (PMC10574539; doi:10.3390/molecules28196990)

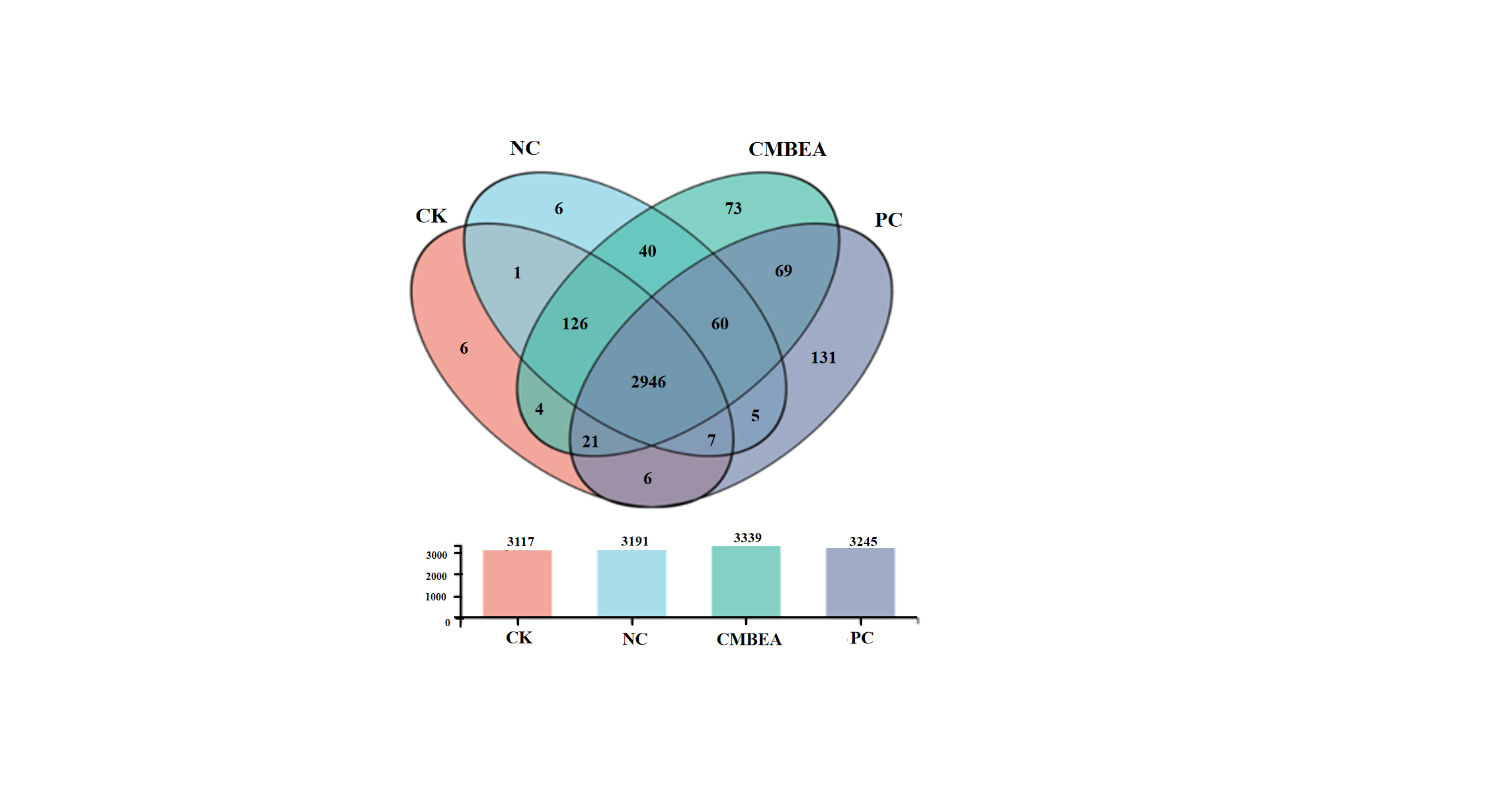

Supplement: Supplementary file 1 [file molecules-28-06990-s001.zip › Additional file/Fig.S1.tif]

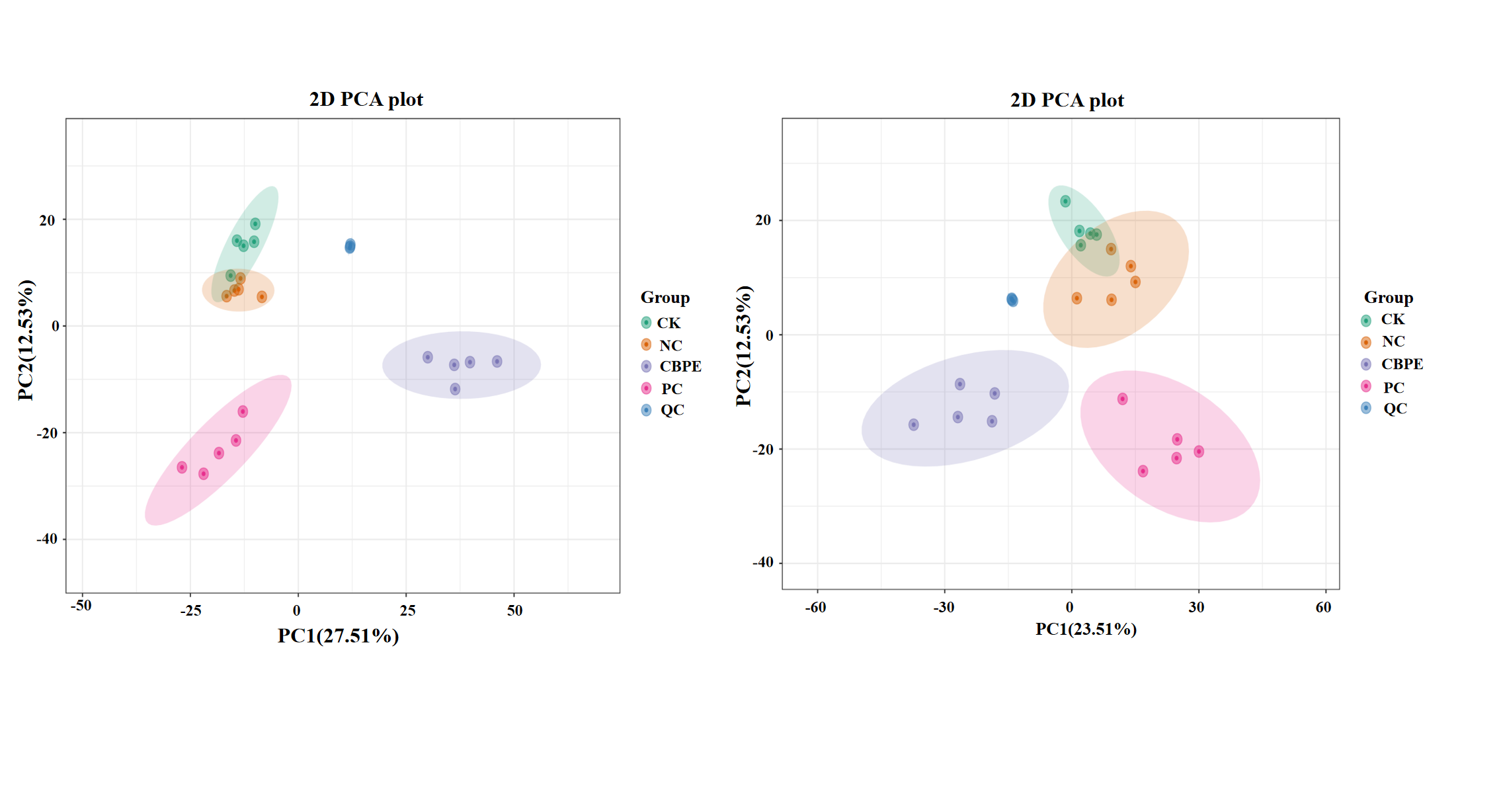

Supplement: Supplementary file 1 [file molecules-28-06990-s001.zip › Additional file/Fig.S2.tif]

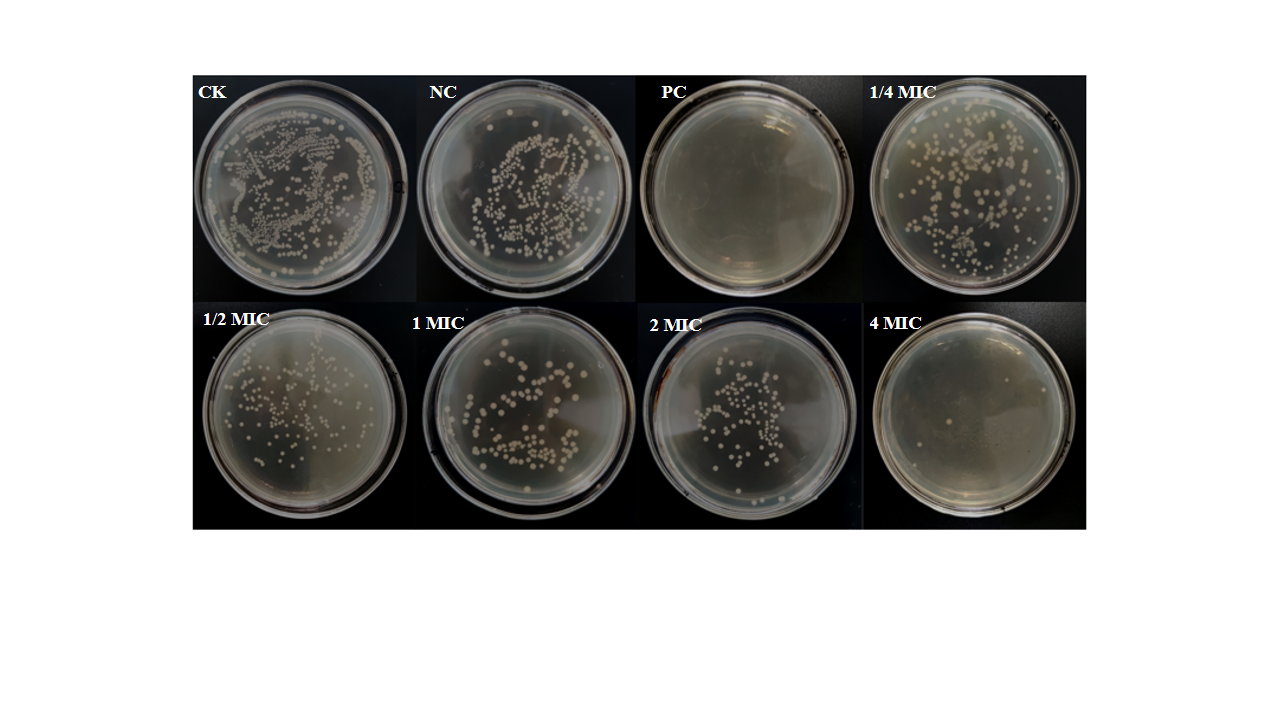

Supplement: Supplementary file 1 [file molecules-28-06990-s001.zip › Additional file/Fig.S3.tif]
